# Caudal regression syndrome from radiology and clinical perspective: A case series and a proposed new integrated diagnostic algorithm

**DOI:** 10.1016/j.radcr.2023.04.015

**Published:** 2023-05-12

**Authors:** Utami Purbasari, Helda Nazar, Faisal Miraj, Dina Aprilia, Wini Widiani, Media Suprihatin, Agnes Nina Eureka

**Affiliations:** a Department of Radiology, Fatmawati General Hospital, South Jakarta, Special Capital District of Jakarta, Indonesia; b Faculty of Public Health, University of Indonesia, Jl. Lingkar Kampus Raya Universitas Indonesia, Depok, West Java, 16424, Indonesia; c Department of Orthopedic and Traumatology, Fatmawati General Hospital, South Jakarta, Special Capital District of Jakarta, Indonesia; d Department of Physical Medicine & Rehabilitation, Fatmawati General Hospital, South Jakarta, Special Capital District of Jakarta, Indonesia

**Keywords:** Caudal Regression Syndrome, Vertebral Agenesis, Sacral Agenesis, Maternal Diabetes, Diagnostic Algorithm

## Abstract

Caudal regression syndrome (CRS) is a rare inherited disorder associated with orthopedic deformities, as well as urological, anorectal, and spine malformations. We present 3 cases of CRS found in our hospital, along with the respective radiologic and clinical findings of the disease. With different problems and chief complaints from each case, we propose a diagnostic algorithm that can be used as a helpful tool in managing CRS. CRS is a complex and rare congenital disorder that affects multiple systems and can result in a range of malformations. The diagnostic algorithm proposed from our findings from 3 CRS cases is important to help healthcare providers identify the types of CRS and apply a more individualized approach to improve the quality of life for the patient.

## Introduction

Caudal regression syndrome (CRS) is a rarely occurring genetic disorder (less than 0.1%-0.5% of newborns) defined as total or partial agenesis of the lower vertebral, including sacral and thoracolumbar spine. This syndrome is part of spinal dysraphism, which is a spinal disorder that occurs due to disruption in the development of the spinal cord. Although there is no clearly determined etiology of CRS, evidence has been found for genetic predisposition, maternal diabetes, and vascular hypoperfusion [1,2].

The CRS has a broad spectrum of systemic anomalies that must be investigated thoroughly at the earliest age [3]. Often reported as sacral agenesis, this rare condition is associated with complex abnormalities of multiple organs such as the genitourinary tract, anorectal organs, and neural system. Without early treatment, these abnormalities may lead to long-term complications. It could be related to vertebral defects, anal atresia, cardiac defects, tracheoesophageal fistula, renal anomalies, and limb (VACTERL) syndrome. We present the radiologic and clinical findings of 3 CRS cases in our center and propose an integrated diagnostic algorithm that clinicians should know what to look for in imaging and how to manage the disease accordingly.

## Case series

### Case 1

**Fig. 1:**
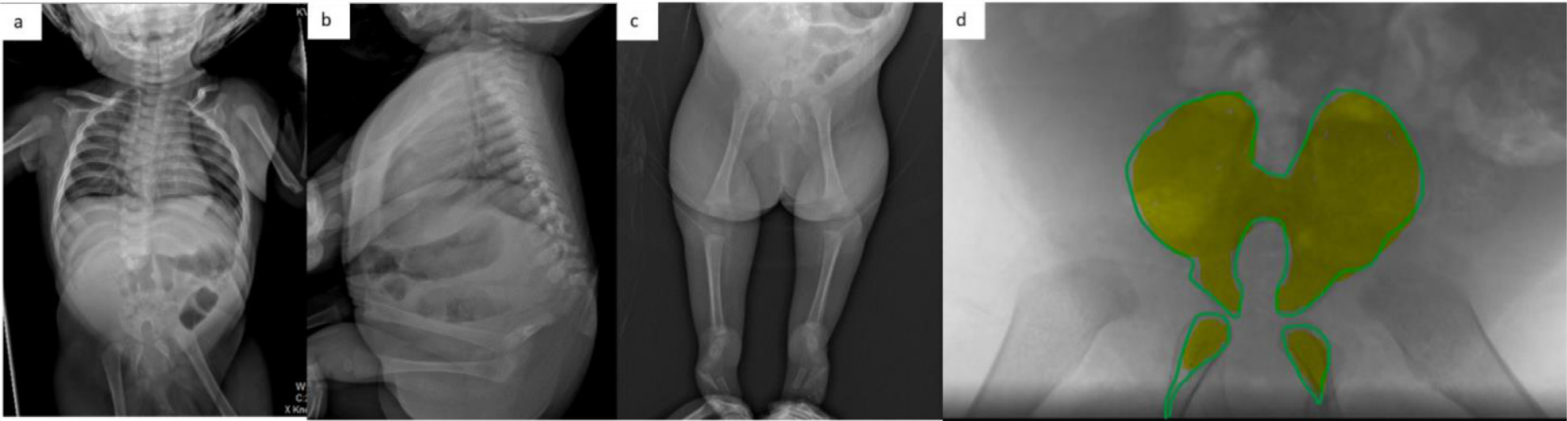
(A-D) Results of the skeletal survey revealing agenesis of lumbosacral vertebrae and visibly hypoplastic pubic and ischium bones. (C) Marked hypoplasia of the extremities and evidence of bilateral CTEV are also seen. (D) A schematic representation of the iliac wing fusion and hypoplastic pelvic.

**Fig. 2:**
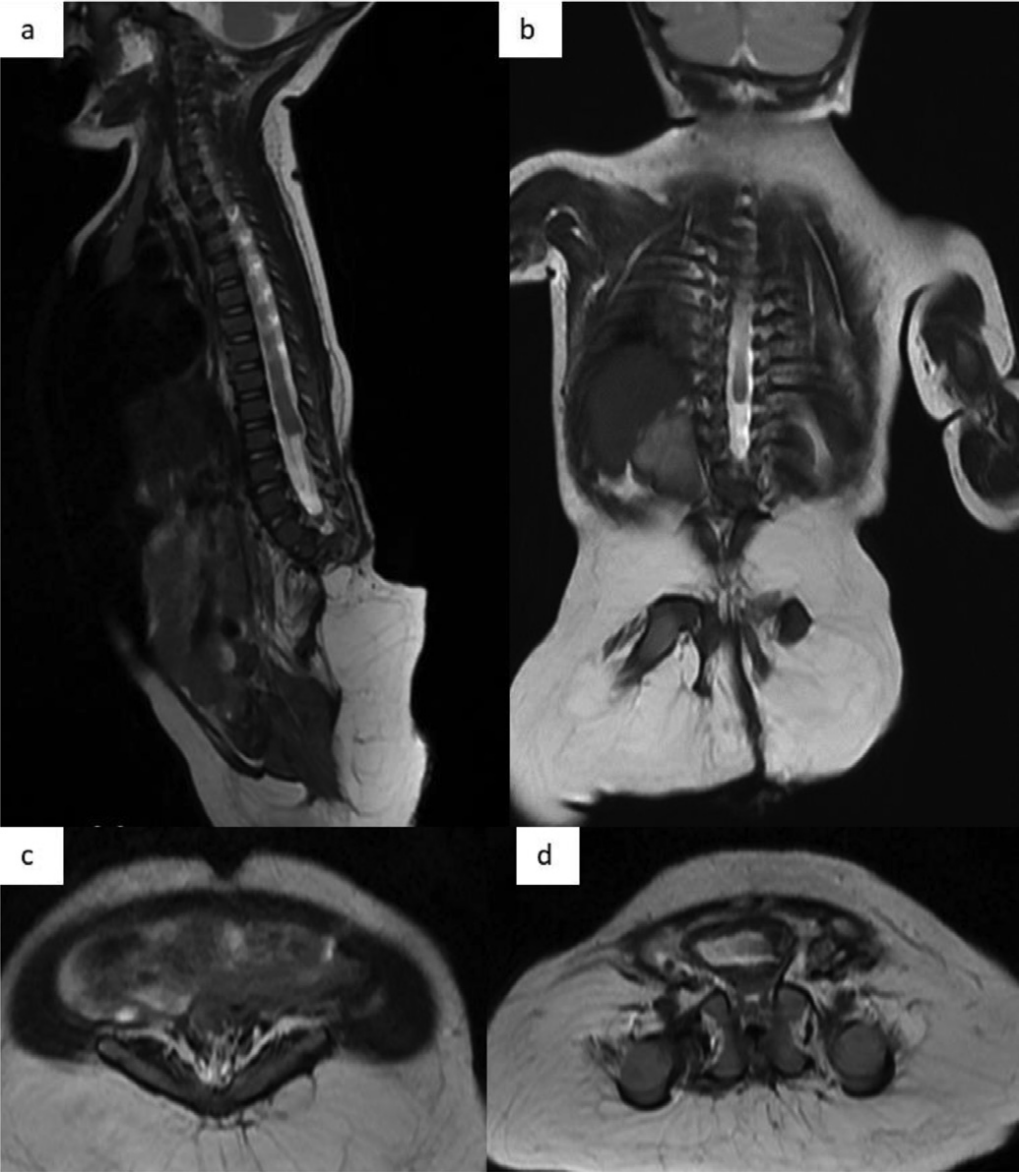
(A, B) An MRI of whole spine at T2 sequences reveals termination of the spinal cord at level of the thoracic 9 vertebra (T9). There is defect at L1 and agenesis of L2-L5 vertebrae, which is replaced with fatty structure. Malformation revealed is identified to be a total sacral agenesis and iliac wing agenesis, consistent with either Type IV (Renshaw) or Type I (Pang) Caudal Regression Syndrome (C, D) An axial view MRI of the pelvic and sacral fusion displays the bladder protruding into the anterior abdominal wall.

A 4-month-old female patient presented with lumps in the back and abnormalities in the lower extremities. She was the youngest of 3 sisters. Her mother had a history of uncontrolled diabetes mellitus during pregnancy. Antenatal ultrasound had revealed abnormalities in her foot. She did not have any history of cigarette smoking or alcohol consumption. A radiographic bone survey was conducted, which revealed sacral agenesis and congenital talipes equinovarus (CTEV). A whole spine MRI showed sacral agenesis type I (Pang) with bilateral iliac wing fusion, agenesis of L2-L5 vertebrae, and a defect at L1 which was replaced with fatty structure. The pubic bone and ischium were hypoplastic. The MRI revealed a blunt and truncated spinal cord at T9, with mild dilatation of the spinal canal filled distally with cerebrospinal fluid. Small kidneys and bladder were also found. The CTEV problems were treated with serial manipulation and casting. However, the parents declined any surgical intervention (Figs. 1 and 2) (Patient Clinical Documentation).

### Case 2

**Fig. 3:**
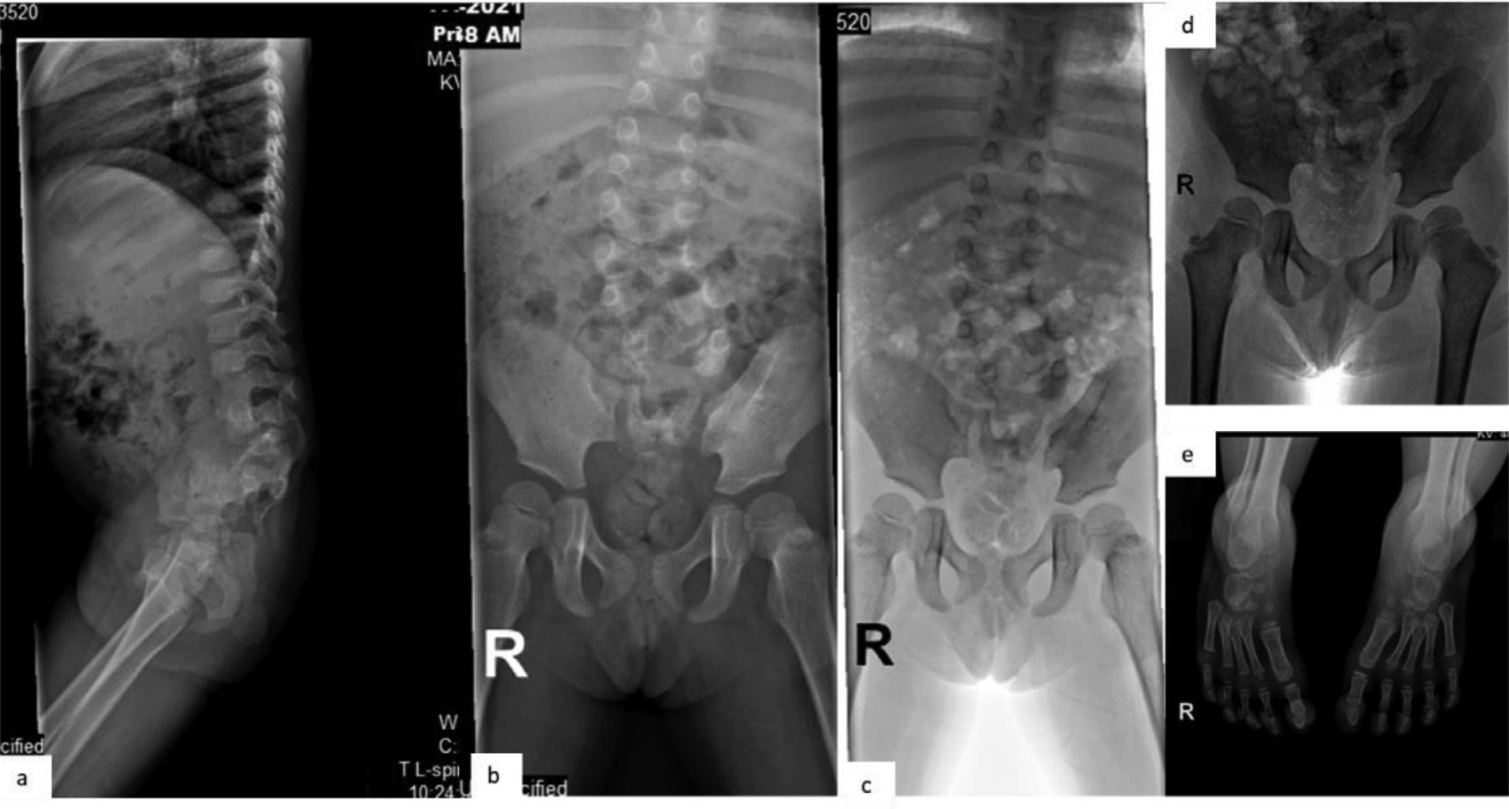
(A and B) Serial radiographs of the spine and pelvic bones display vertebral deformity with hemivertebrae in the lumbar segments and partial agenesis of the sacral bones, consistent with Pang Type IV as seen more clearly in the inverse technique (C and D). The iliac wings and pubic bones are present. (E) Bilateral foot deformity due to CTEV is also visible.

**Fig. 4:**
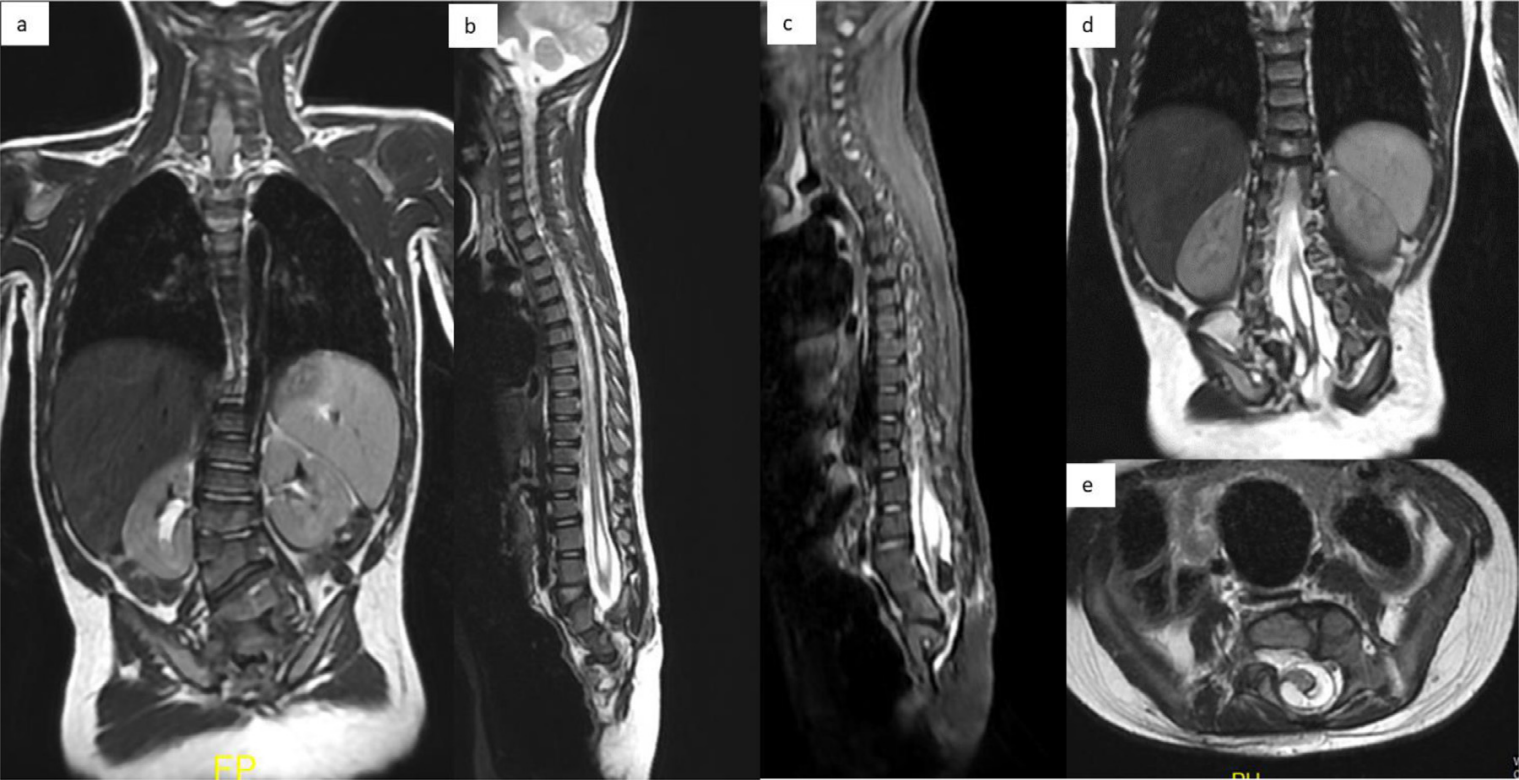
(A-E) An MRI of the entire spine using T1-T2 sequences reveals a failure of lumbar segmentation or the presence of hemivertebrae (A) and partial sacral agenesis (B and C) consistent with Pang type IV. This malformation is indicated by the low-lying conus and sign of syringomyelia (B-D). In this case, the retained medullary cord with the conus located below the lumbar was found to be attached to fatty structure (D and E).

A 3-year-old female patient presented with scoliosis, bilateral clubfeet, delayed gross motor skills, and urinary problems. Other findings were unremarkable. She was the youngest of 3 siblings, born full term by vaginal birth with normal weight and development, but with gross motor disturbances. A radiographic bone survey and MRI showed sacral agenesis, lumbar segmentation failure or hemivertebrae, and extremity deformity (Fig. 3). The MRI also showed a bifid spine, dilated distal spinal canal with tethered cord, and syringomyelia, with conus medullaris at the level of L5 showing failed regression with tethered cord. The sacral formation was consistent with Pang type IV sacral agenesis. The patient underwent neural surgery to correct the tethered cord with laminectomy to free the phylum terminale attached to the fat structure near the lumbar spine (Fig. 4). The medulla was separated from the fat structures for decompression, and the patient was referred to an orthopedic surgeon for serial manipulation and casting, followed with bilateral achilles tendon lengthening (ATL) for the clubfeet. The patient also underwent a urodynamic study and received bladder management therapy along with physical rehabilitation.

### Case 3

**Fig. 5:**
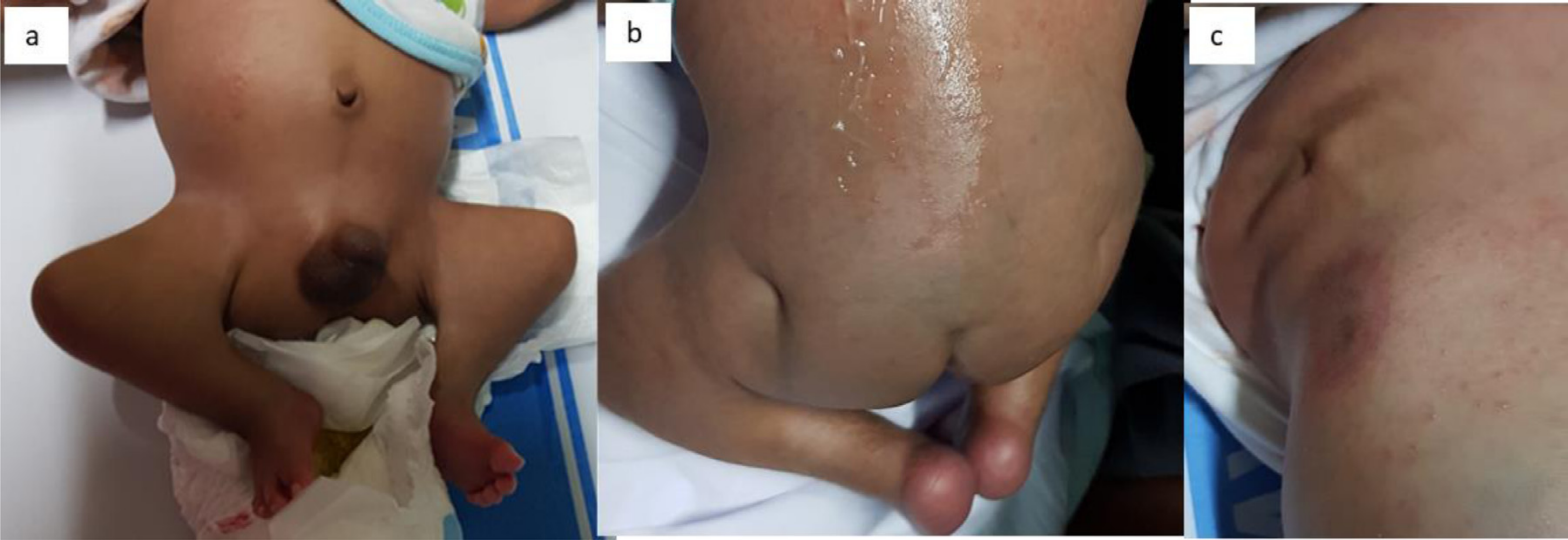
(A) A 3-month-old baby with hips in flexion and abduction position when the patient was in supine position, and (B) with deformity and dimple in the hips, suggesting a DDH (developmental dysplasia of the hip). (C) At lateral position (craniocaudal view) there was also bulging and dimple at the upper backbone due to deformity of the thoracic vertebrae.

**Fig. 6:**
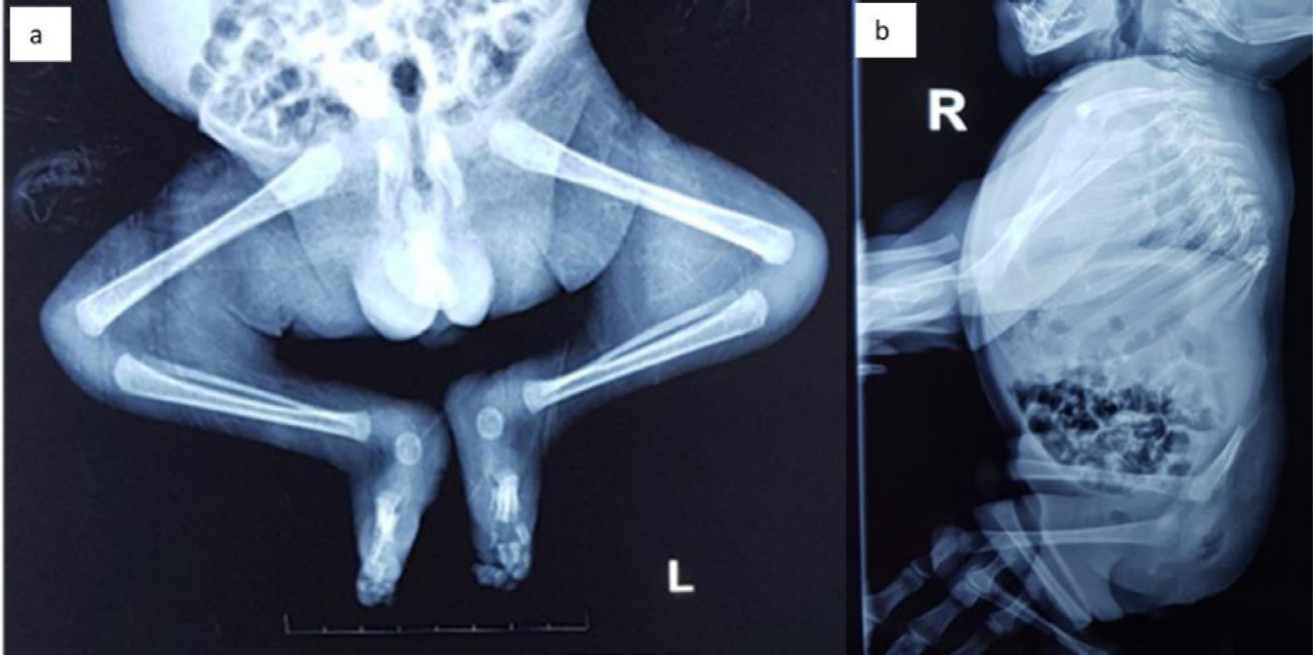
(A) Radiography of whole spine and lower extremities showed hypoplastic ilium and ischium bones. The flexion position was due to the stiffness in the lower extremities, with bilateral clubfoot deformity. (B) In lateral view, signs of partial sacral agenesis and underdevelopment of the lower thoracic, lumbar spine, and sacrum were visible.

**Fig. 7:**
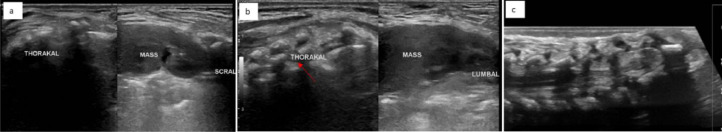
Due to parents’ refusal to undergo an MRI, a spinal ultrasound was performed. Results showed the absence of lumbar and sacral bones, which were replaced by a soft tissue structure of hypoechoic mass, resembling non ossification lumbosacral (A and B). The conus medularis was found to terminate at a high level in the thoracic region (indicated by red arrow in B). Figure 7C shows a similar case from a spinal ultrasound, in which the conus medullaris extends to the level of T12. Below this level, no vertebral bodies or spinal canal were detected. (Note: Figure 7C is reproduced from Janice et al., 2018 https://www.eurorad.org/case/14829).

A 3-month-old male infant from a rural area, the youngest of 3 children born to an elderly mother, with no history of congenital malformations. Physical examination reveals deformities of the lower extremities, including hips in a flexed and abducted position, visibly flat buttocks, narrow hips, lower limb atrophy, and hip dysplasia. Bilateral dimples in the hips and a dimple at the spinal column were also observed, suggesting developmental dysplasia of the hip (DDH) and vertebral and hip deformities (Figs. 5 and 6). Radiographic examinations showed agenesis of lumbar vertebrae (L1-L5) and sacral agenesis, consistent with type I Pang's classification. A spinal ultrasound revealed absence of the lumbar and sacral bones, replaced by soft tissue structures and a high-level conus medullaris stopping at the thoracic region (Fig. 7). However, the cartilaginous of the femoral head could not be evaluated through a hip ultrasound. The parents declined further treatment, including an MRI.

## Discussion

Caudal regression syndrome is rare congenital malformation associated with severe skeletal deficiencies of the vertebral or sacrococcygeal bones, resulting in complex anomalies in the gastrointestinal, neural, orthopedic, and genitourinary system [2,4]. This syndrome is characterized by complex dysraphism with aberrations in gastrulation and failure of notochord formation. The notochord is a key structure in fetal development, and its formation occurs during gastrulation, around the third week of gestation, as cells divide from the primitive streak. This defect has been attributed to the failure of neuralization at day 28 of fetal development. The notochord develops by cephalad elongation, which is paralleled by the development of the neural plate. It stimulates the condensation of paraxial mesoderm into segments called somites. Within these somites, the mesoderm further condenses to form the sclerotomes, which will become the vertebral bodies and the costae. The formation of somites is closely linked to the development of the heart, making other organs that develop at the same time as the vertebral bodies vulnerable to anomalies in Caudal regression syndrome [2]. Family history and maternal diabetes are 2 known risk factors for caudal regression syndrome. The prevalence of CRS in infants of diabetic mothers can reach up to 1 in 350 live births. A dominantly inherited defect in the HLXB9 gene has been described as Currarino syndrome which may also be related to CRS. Other possible risk factors and pathomechanisms include maternal exposure to cocaine or alcohol, a vascular steal theory, fetal hypoxemia, and amino acid imbalances [5].

Clinical manifestations that may be observed include short intergluteal folds, flat buttocks, narrow hips, lower limb atrophy, hip dysplasia, and congenital talipes equinovarus (CTEV). Genitourinary abnormalities include horseshoe kidney, cystic dysplasia, neurogenic bladder, and Müllerian duct abnormalities, while gastrointestinal abnormalities may include duodenal atresia, imperforate anus, and intestinal malrotation [5]. Detecting sacral agenesis and its associated malformations during prenatal, neonatal, and early life stages is crucial for identifying risks, preventing complications, and managing long-term outcomes through multidisciplinary approaches. The goal is to detect the risk, prevent complications and address the long-term outcomes which may require multidisciplinary approaches [6].

Prenatal diagnosis of CRS can be challenging, especially in milder forms. Maternal ultrasound is a sensitive tool for the prenatal diagnosis, and fetal MRI can be used to confirm this complex syndrome. The diagnosis of fetal CRS requires antenatal ultrasound. Findings of hypoplasia of the lower extremities and sacral or lumbosacral agenesis are correlated with the diagnosis of CRS. In the first trimester, the fetal crown-rump length (CRL) of CRS fetuses is typically shorter per week than that of normal fetuses, according to reports. It is difficult to diagnose sacral agenesis on ultrasound because the sacrum is not sufficiently calcified before 19 weeks of pregnancy [7], [8]–9]. Antenatal diagnosis can be suspected in the first trimester and confirmed in the second trimester of gestation. In the first trimester, lumbosacral agenesis will result in a decrease of cranio-caudal length. Fetal MRI will confirm the diagnosis and could determine the level of the terminal medullary cone, which will be a major prognostic factor. The spinal cord is usually dysplastic, and the terminal cone is too high in cases with poor prognosis. The prognosis of the CRS depends on the prognosis of the associated malformations, the level of the medullary cone and the impact of diabetes to fetal [10]. Fetal MRI should be considered in obese patients and in patients with oligohydramnios [10].

After birth, other means of diagnostic imaging in cases of CRS includes ultrasonography, bone survey, and CT scan. Spinal ultrasound, abdominal ultrasound and head ultrasound were performed to confirm the abnormalities, detect the cord termination and genitourinary abnormalities. Skeletal survey or radiographic examinations were performed to visualize the type of CRS. CT Scan and MRI are important to visualize the internal organs, spinal cord and reconstruct the bone malformations. Sagittal T1 and T2 weighted spin-echo, sagittal short-tau inversion recovery (STIR), axial T1/T2 weighted images, and sagittal echo gradient (GRE) images of the lumbosacral and pelvic regions are recommended for protocol in dealing with CRS. Some cases need orthopedic surgery to prevent instability or ability to walk or sitting. We should perform urodynamic study or VCUG (*Voiding cystourethrography*) to detect urogenital malformation and prevent the risk of renal damage [11].

The classification proposed by Pang et al [12]. is a popular method of classification used in the diagnosis of CRS. In Pang types I and II, there is complete absence of sacrum accompanied by or without lumbar vertebrae agenesis respectively. In type III, S1 is present but the lower sacral segments are missing to varying degrees. Type IV consists of various forms of hemisacrum and type V includes coccygeal agenesis cases [13]. In our cases, we diagnosed Pang Types I and IV of CRS. However, the exact incidence of each type is not well-established due to the rarity and wide variability of the disorder. Some literature suggests that partial sacral agenesis with symmetrical defects of the ilia and first sacral vertebrae is a common form of CRS [14].

The management of CRS is highly individualized for each case and must include a wide variety of interventions to address the full spectrum of possible anatomical abnormalities. The early CRS interventions are essentially needed and considered as “life saving” for the associated anomalies in pulmonary, gastrointestinal, or urorectal problems, including tracheoesophageal fistula, cloacal anomaly, omphalocele, bladder exstrophy, and imperforate anus [13].

The next special attention being the orthopedic abnormalities as the core problems of the disease. The major orthopedic problems in CRS are spinal deformities, spino-pelvic instability, hip dislocation and contractures, knee contractures, and foot deformities. The management varies from the conservative to surgery by deformity correction and stabilization. Spinopelvic stability is essential for a stable sitting and ambulation. The patients with unstable spino-pelvic structure must be observed for the progressing spinal deformity and instability (indicated by worsening of the body posture) with the associated trunk shortening and abdominal collapse which further cause multiple organ problems ranging from the cardiorespiratory, gastrointestinal, and the urogenital. Thus, the management of Pang type I CRS is more likely to provide support and to improve the residual function than to correct deformities [4]. Spinal brace, sub-throchanteric amputations with pelvic thoracic prosthesis, or spino-pelvic fusion without or with multilevel pedicular screw fixation were options reported in various literatures [3,^5^,^12^,^15^,16]. The latest was associated with an increased quality of life by improving core body stability and spinal sagittal balance [4,14]. The spino-pelvic fusion may improve intestinal transit, posture, sitting position, and allow stretching contractures that may be resulted from a more effective physiotherapy in a stable spino-pelvic condition. Thus, although a more aggressive approach with limb amputation is aiming to a better sit and more effective walk (with hands), the limb salvage procedures are generally benefits from a better body image and proprioceptive. For the progressing deformity particularly in preadolescent ages, bracing can be an option. However, it may be ineffective with the presence of spino-pelvic instability. On that case, scoliosis correction with spinal and spino-pelvic fusion can be considered [4].

Neurosurgical management is generally less urgent and may have a limited role because the neurologic deficits are usually permanent due to the irreversible primary pathology. Untethering procedure is indicated primarily for patients with the conus below L1 which is more likely to suffer from progressive neurological deficits due to the tethering cord. In patient with conus above L1, the untethering procedure is unlikely to benefit because the neurological symptoms are static in nature. Moreover, decompression is also indicated in the presence of dural or bony stenosis which may also cause neurological problems [2,13].

The tethered cord syndrome (TCS) is defined as a stretch-induced functional disorder of the spinal cord with its inferior part anchored (tethered) by inelastic structures [17]. In our second case, the conus was the regression failure types and the consequent of this low-lying conus is concerning for tethering [17]. Thus, the untethering procedure seems to be beneficial in this patient in order to improve the neurologic functions. The additional laminectomy was indicated due to the evidence of nerve compression on the level of lumbar vertebrae L5.

The management of lower extremity deformities is determined by how much these deformities are contributing to postural difficulties or mobility restrictions. Hip contractures are most commonly managed conservatively with serial stretching and surgical release (in a more severe form). Controversies exist in the management of the dislocated hip. Some literatures suggest a more conservative way due to the risk of aseptic femoral head necrosis after reduction which leads to painful hip and the reduction is only suggested in the patient with ambulatory potential. However, a persistent dislocation may cause pressure sores, gait deterioration, and increased pelvic obliquity which leads to further spinal imbalance. Thus, the indication of hip reduction can be widened considering the long-term consequences of hip dislocation [4].

The management of knee contractures are not only essential in a presence of lower motor function but also beneficial to improve sitting in non-ambulatory patients. The management varies from serial stretching, bracing, surgical contracture release, up to amputation/disarticulation, depends on the severity of contracture and the presence of neurologic function. Lastly, foot deformities in the form of equinovarus, equinovalgus, and calcaneovalgus may be treated conservatively by serial manipulation and casting with or without additional surgical procedures such as releases and arthrodesis. The aims of foot deformities correction are to improve mobility in ambulatory patients and shoe-wearing ability in the nonambulatory patients. The serial manipulation and casting as described by Ponseti [18] works well in one of our cases, while another needed additional surgical release by Achilles tendon lengthening to correct the residual equinus after the initial series of manipulation and casting [4].

Urodynamic testing is a crucial component to the evaluation and identification of the neurogenic bladder lesion associated with SA. These studies can provide a comprehensive evaluation of lower urinary tract function or dysfunction, and in these situations, they offer insight and assist to guide management toward maintaining low-pressure safe storage bladders with adequate compliance to preserve the upper urinary tract. For example, this data may help determine if a patient's current bladder management is appropriate. This information can guide treatment–whether to adjust medical management or prompt consideration for surgical intervention. The present institutional experience has been limited by the amount of available serial UDS, due to deferred or missing documentation, and varied practice patterns among healthcare teams. For patients diagnosed with SA at the present institution, UDS is performed to establish a baseline and then at intervals based on changes in clinical picture [7].

**Fig. 8:**
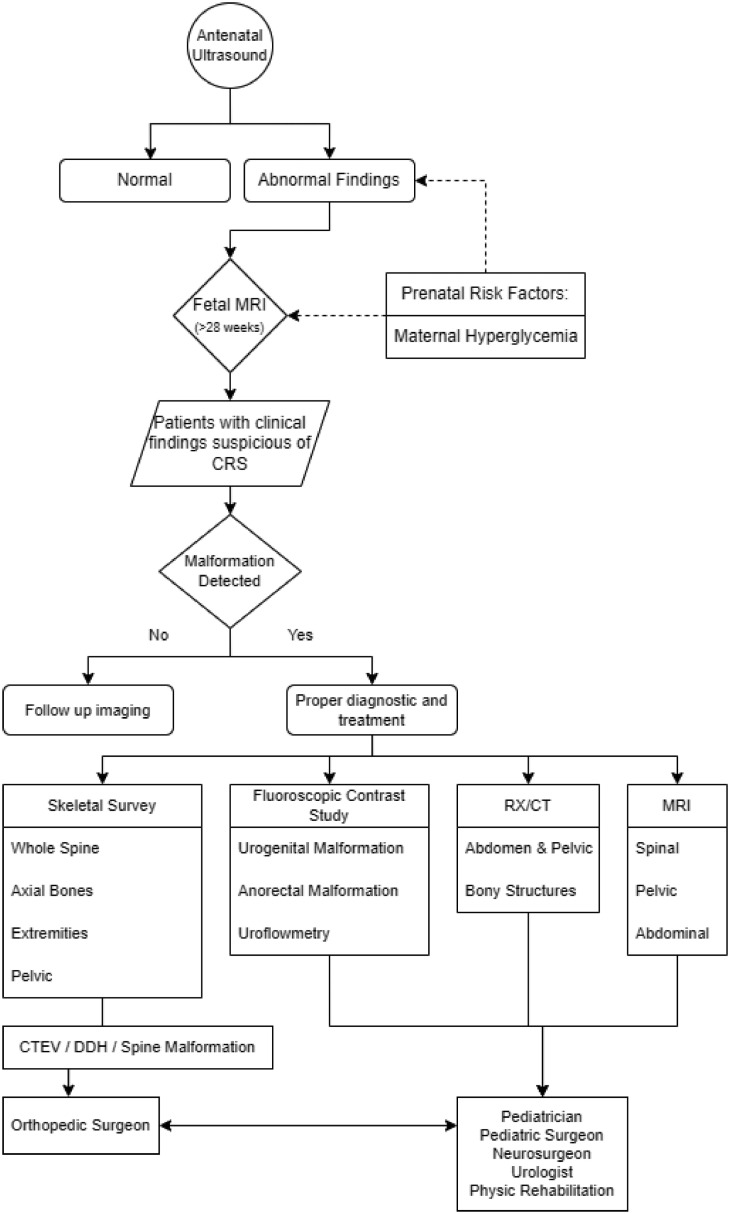
Proposed diagnostic algorithm.

Understanding the high complexity and the multiple system involved in CRS and CRS as rare inherited disorder may allow a better multidisciplinary approach to the management of patients with CRS. An integrated diagnostic algorithm is very important to identify the specific types of CRS and help healthcare providers to manage the patient to avoid unnecessary examination and radiation and improve the patient's quality of life. We propose a diagnostic algorithm (Fig. 8) aimed at increasing the awareness of clinicians that found a possibility of a CRS case, to initiate a comprehensive multidisciplinary approach for an accurate diagnosis and effectively treat the patient in a comprehensive and holistic manner. These clinicians include radiologists, orthopedic surgeons, obstetricians/gynecologists, pediatricians, urologists, neurosurgeons, cardiothoracic surgeons, physical therapists, and orthotists.

## Conclusion

Caudal Regression Syndrome is rare congenital malformations associated with agenesis of vertebral or sacrococcygeal bones, which can result in complex anomalies in gastrointestinal, neural, orthopedic, and genitourinary system. Treatment requires a multidisciplinary approach. A diagnostic algorithm for CRS is crucial to understand the types of CRS and assist radiologists and clinicians in managing patients, to avoid unnecessary examinations, radiation, and improve the patient's quality of life. It should be noted that the aim of rehabilitation is not to correct all deformities, but to increase the functionality in everyday life. Some patients with a bent knee position and webbing between the thigh and calf may require knee arthrotomy to allow mobility and a better sitting position. Amputation followed by wheelchair or prosthetic mobility is the preferred treatment, but some patients are known to adapt well and mobilize using their hands to this condition. There are no cognitive deficits associated with this disorder. It is important to note that a delay in diagnosis of CRS can result in progressively deterioration, particularly an increased risk of urinary tract infections and other complications [7].

## Patient consent

The authors of this case series titled *“Caudal Regression Syndrome from Radiology and Clinical Perspective: A Case Series and A Proposed New Integrated Diagnostic Algorithm”* state that informed consent for the publication of this case series was obtained from the legal guardians of the pediatric patients involved. All patient identifying information has been redacted to protect the privacy of the individuals involved.

## References

[1] Bouchahda H, El Mhabrech H, Hamouda H Ben, Ghanmi S, Bouchahda R, Soua H (2017). Prenatal diagnosis of caudal regression syndrome and omphalocele in a fetus of a diabetic mother. Pan Afr Med J.

[2] Akhaddar A. (2020). Caudal Regression syndrome (Spinal Thoraco-lumbo-sacro-coccygeal Agenesis). World Neurosurg.

[3] Albarayak MBBA, Yunus A, Temel T, Balioglu K, Yildirim M (2016). Caudal Regression Syndrome (Sacral Agenesis) with associated anomalies. J Turkish Spinal Surg.

[4] Altaf F, Hakel W, Sivaraman A, Noordeen H. (2008). Orthopaedic management of caudal regression syndrome. Eur J Orthop Surg Traumatol.

[5] Kylat RI, Bader M. (2020). Caudal regression syndrome. Children.

[6] Cho PS, Bauer SB, Pennison M, Rosoklija I, Bellows A L, Logvinenko T (2016). Sacral agenesis and neurogenic bladder: Long-term outcomes of bladder and kidney function. J Pediatr Urol.

[7] Sharma S, Sharma V, Awasthi B, Sehgal M, Singla DA. (2015). Sacral agenesis with neurogenic bladder dysfunction - a case report and review of the literature. J Clin Diagn Res.

[8] Negrete LM, Chung M, Carr SR, Tung GA. (2015). In utero diagnosis of caudal regression syndrome. Radiol Case Rep.

[9] Temizkan O, Abike F, Ayvacı H, Yılmaz A, Demirag E, Sanverdi İ. (2013). Prenatal diagnosed caudal regression syndrome. Open J Obstet Gynecol.

[10] Bouchahda H, Mhabrech H el, Hamouda H ben, Ghanmi S, Bouchahda R, Soua H (2017). Prenatal diagnosis of caudal regression syndrome and omphalocele in a fetus of a diabetic mother. Pan Afr Med J.

[11] Boruah D, Dhingani D, Achar S, Prakash A, Augustine A, Sanyal S (2016). Magnetic resonance imaging analysis of Caudal Regression Syndrome and concomitant anomalies in pediatric patients. J Clin Imaging Sci.

[12] Lee J, Pang D, Wang K, Rocco C, Pang D, Rutka J (2020). Textbook of pediatric neurosurgery.

[13] Lee JY, Shim Y, Wang KC. (2021). Caudal agenesis: understanding the base of the wide clinical spectrum. J Korean Neurosurg Soc.

[14] Dayasiri K, Thadchanamoorthy V, Thudugala K, Ranaweera A, Parthipan N (2020). Clinical and radiological characterization of an infant with Caudal Regression syndrome type III. Case Rep Neurol Med.

[15] Banta J, Nichols O (1969). Sacral agenesis. J Bone Joint Surg Am.

[16] Dumont C, Damsin J, Forin V (1993). Lumbosacral agenesis. Three cases of reconstruction using Cotrel-Dubousset or L-rod Instrumentation. Spine (Phila Pa 1976).

[17] Proctor MR, Kliegman RM (2020). Nelson textbook of pediatrics.

[18] Ponseti IV. (1996).

